# COVID-19 related decline in cancer screenings most pronounced for elderly patients and women in Germany: a claims data analysis

**DOI:** 10.1007/s00432-022-04433-z

**Published:** 2022-11-27

**Authors:** Jennifer Muschol, Cornelia Strauss, Christian Gissel

**Affiliations:** 1 Department of Health Economics, Justus Liebig University Giessen, Klinikstraße 29, 35392 Giessen, Germany; 2 Department of Behavioral and Institutional Economics, Justus Liebig University Giessen, 35394 Giessen, Germany

**Keywords:** Claims data analysis, COVID-19, Cancer screening, Gender inequalities, Age inequalities, Public health

## Abstract

**Purpose:**

This study aimed to analyze the utilization of cancer screenings in Germany before and during the COVID-19 pandemic in 2020. The objective of the analysis was to identify the population at particular risk and to derive recommendations for the future use of resources to prevent long-term deteriorations in health outcomes.

**Methods:**

The analysis was conducted based on claims data of all preventive health services for 15,833,662 patients from the largest statutory health insurance fund in Germany. Utilization of general female cancer screening, general male cancer screening, general health checkup, colorectal cancer screening stool test, colorectal cancer screening consultation, colonoscopy, skin cancer screening, and mammography screening was compared before (2017–2019) and during (2020) the pandemic.

**Results:**

Data of a total of 42,046,078 observed screenings showed that the utilization of the individual screenings developed differently, but that the overall utilization decreased significantly by 21.46% during the COVID-19 pandemic (*p* < 0.001). At the same time, no catch-up effects were detected for total screenings throughout the entire year 2020. The highest decline in screenings was found for the elderly (*p* < 0.001) and women (*p* < 0.001).

**Conclusion:**

Because the elderly are at higher risk for cancer, the omission of early detection might lead to higher treatment costs, reduced quality of life, and higher mortality. In addition, women's medical care in particular has been negatively affected, for example, by the interruption of mammography screenings and the lack of catch-up effects. Therefore, resources must be targeted to reduce burdens on health outcomes and public health in the long term.

## Background

Noncommunicable diseases such as cancer and cardiovascular diseases have a negative impact on public health by causing approximately 71% of deaths worldwide each year. Moreover, they are associated with reduced quality of life and lower life expectancy, as well as economic burdens in the form of rising treatment costs and declining productivity (Dzau et al. 2017; World Economic Forum 2017; World Health Organization 2021). Preventive health services are an important component of public health as early detection and treatment of noncommunicable diseases such as cancer and their precursors can reduce incidence, disease severity, and mortality (World Health Organization. Regional Office for Europe 2020). As a result, countries around the world, including Germany, offer screening programs that are legally regulated. For example, since 2008, a skin cancer screening program has been offered free of charge for patients with statutory health insurance (SHI) in Germany. The program’s effects were desirable from a public health perspective: since its introduction, an increased incidence of skin cancer has been observed, but the cases detected were mainly in earlier stages of the disease (Girbig et al. 2021). Especially in malignant melanoma, early diagnosis and treatment are crucial as it has a direct impact on the survival rates (Girbig et al. 2021; McBain et al. 2021). Early diagnosis also plays an important role in successful treatment for other types of cancer, underlining the relevance of preventive health services for health-care systems (World Health Organization. Regional Office for Europe 2010).

The screening program in Germany comprises a total of ten different services and primarily focuses on the early detection of cancer. Only the screening for colorectal cancer, cervical cancer, and breast cancer is organized and eligible patients are invited based on a register, while other screenings can be utilized opportunistically. Screening utilization varies in Germany with respect to the individual examinations, sex, and age. For example, more than 50% of women younger than 70 years make use of female cancer screening every 3 years. Male cancer screening, on the other hand, is only used by up to 35% in at least 5 out of 10 years. In total, participation rates were relatively constant before the COVID-19 pandemic (Tillmanns et al. 2021).

With the spread of the COVID-19 pandemic since March 2020 (World Health Organization. Regional Office for Europe 2022), however, international health-care systems have been disrupted (World Health Organization 2020). In addition to the direct medical impact from infections and associated mortality, the pandemic has led to widespread limitations in medical services (Wang et al. 2020). For example, a change in the utilization of outpatient services such as for cancer screenings was observed internationally (Chen et al. 2021; Damerow et al. 2020; Doubova et al. 2021). The postponed or canceled screenings, however, could be linked to the risk of delayed diagnosis and, thus, more severe disease progressions and the duration of suspended screenings and potential catch-up effects will have a strong impact on long-term death rates (Alkatout et al. 2021; Blumen et al. 2016; Burger et al. 2021; Duffy et al. 2022; Kregting et al. 2021; Maringe et al. 2020; Yong et al. 2021).

Compared to international findings, scientific publications on the utilization of preventive health services during the pandemic based on German data are limited (Alkatout et al. 2021; Mayo et al. 2021). However, to contain the impact of postponed and canceled screenings in Germany and to best prevent poorer health outcomes, increased mortality and rising health-care expenditures, a targeted use of limited health-care resources is essential in the long term. Prioritization of particularly vulnerable patient groups is only possible if differences in utilization are known, which can only be derived from examining trends across all preventive health services offered in a country and goes beyond simply analyzing individual screenings.

This explorative study attempts to fill the existing research gap by analyzing the utilization of all preventive health services and cancer screenings legally regulated for German patients using claims data from 15,833,662 patients in the SHI before and during the COVID-19 pandemic to identify the population at particular risk and to derive recommendations for the best possible use of resources in future preventive health programs. In addition, the change in outpatient reimbursement of screenings before and during COVID-19 will be compared in the form of a health economic analysis as part of this study.

## Methods

### Study design and data source

The retrospective claims data analysis was based on national claims data from adult persons who were insured at the Allgemeine Ortskrankenkasse (AOK) in Germany between 2017 and 2020. AOK consists of 11 regional health-care funds and together they represent the largest SHI fund in Germany. With 27 million insured persons, around one-third of the entire German population is covered by AOK (AOK-Bundesverband 2022; Schulz et al. 2020).

The data analyzed in the study were provided by the AOK Research Institute (WIdO) and served the primary purpose of the reimbursement of services between providers and payers. WIdO processed the requested data on the basis of a predefined study protocol and made them available for the purpose of the study. The study protocol was prepared in accordance with the guideline for Good Practice of Secondary Data Analysis (GPS) (Swart et al. 2015). In addition, the Consensus German Reporting Standard for Secondary Data Analyses, Version 2 (STROSA 2) was used as a guidance for the reporting of the study, as it was developed especially for the particular requirements of German claims data analyses (Swart et al. 2016).

The anonymized data set contained the claims data of the fee schedule items (GOP) for all preventive health services legally regulated for adult persons with SHI coverage in Germany. These included GOP 01730, 01760, and 01761 for general female cancer screening, 01731 for general male cancer screening, 01732 for general health checkup, 01734 and 01738 for colorectal cancer screening stool test, 01740 for colorectal cancer screening consultation, 01741 for colonoscopy, 01745 and 01746 for skin cancer screening, and 01750 for mammography. Beyond these GOPs, the data set included claims data for regionally agreed services for the listed screenings. Only claims data for early detection of abdominal aortic aneurysms were omitted because a complete data set for this preventive health service was not available for the observation period of the study. The term “screening” will be used synonymously for all preventive health services considered in the study.

The data set comprised the aggregated number of claims data on a monthly basis from January 2017 to December 2020, specified by the age and sex of patients eligible for the respective examinations. Age categories were formed on the age calculated at the end of December 2020. Only the claims data of AOK were used, and no further data linkage was performed. Table 1 provides an overview of the data obtained.

**Table 1:** Overview of the screenings and eligible patients considered

GOP	Type of examination	Sex	Age^a^
01730, 01760, 01761	General female cancer screening	Female	≥ 25 years
01731	General male cancer screening	Male	≥ 50 years
01732	General health checkup	Female and Male	≥ 40 years
01734, 01738	Colorectal cancer screening stool test	Female and Male	≥ 55 years
01740	Colorectal cancer screening consultation	Female and Male	≥ 55 years
01741	Colonoscopy	Female and Male	≥ 60 years
01745, 01746	Skin cancer screening	Female and Male	≥ 40 years
01750	Mammography screening	Female	≥ 55–69 years

^a^The age groups were raised by 5 years compared to the actual eligibility for the respective preventive health services, as the age of the patients was calculated at the end of 2020

### Sample and population

The study included all AOK insured persons who were 25 years and older, eligible for the individual screenings based on their age and sex, insured in all quarters from 2017 to 2020, and who did not die in the fourth quarter of 2020. Patients that were participating in a primary physician model were excluded from the data set for data protection reasons. An a priori sample size calculation was not performed due to the explorative study design.

### Legal basis and data protection

Claims data are transferred to the AOK according to § 295 of the German Social Code, Book V. The transfer of social data such as claims data for the purpose of research is regulated in § 67b and § 75 of the Social Code, Book X. As the data holder, the WIdO has consented to the provision of the data for the exclusive purpose of this study, taking into account data protection measures. Because the data set submitted by the WIdO only contained the aggregated number of screenings, the anonymized data did not allow any conclusions about individual persons. For this reason, no informed consent was required from the individuals included in the data set. Furthermore, according to the GPS guideline, the consultation of an ethics committee is not required for analyses of claims data (Swart et al. 2015).

### Data processing, statistical analyses, and health economic analysis

During data preparation, the age categories 25–39 years, 40–59 years, 60–79 years, and  > 80 years from a study by Kremer and Thurner (2020) were used to further summarize the age of the patients (Kremer and Thurner 2020). In addition, to compare utilization of screenings before COVID-19 and during COVID-19, the number of claimed screenings in the years 2017, 2018, and 2019 were averaged. This approach was intended to compensate for potential bias in previous years and to provide an approximation of the actual effects of the pandemic. Thus, the average utilization values from 2017 to 2019 were set as the time before COVID-19. Although the COVID-19 pandemic was declared as such not before March 2020, the first cases were reported in January 2020, which is why the values from 2020 were declared as the time during COVID-19 in the context of this study for the simplicity of the calculation.

The arithmetic mean and standard deviation (SD) of the change in monthly screening utilization before and during the COVID-19 pandemic were calculated using the observations from each GOP differentiated by sex and age category. Due to the sex- and age-based eligibility of the screenings, the total number of data points for the calculation of the mean and SD of each screening was 37 per month. Concerning the evaluation of differences in the utilization of screenings between women and men, only screenings that were available to both sexes were considered, resulting in a calculation of the mean and SD from a total of 14 data points per month per sex. Statistical analysis included the conduction of the binomial test to examine whether utilization changed significantly during the COVID-19 pandemic compared with the time before COVID-19. For this purpose, the respective proportion of utilized screenings during COVID-19 was compared with the proportion of utilized screenings before COVID-19 based on the number of insured persons that did not change during the observation period. This analysis comprised the individual GOPs, sex, and age of the patients. In addition, the independence of screening utilization before and during COVID-19 over the time course was tested using Pearson’s Chi-square test. This analysis was furthermore extended by distinguishing between sex and age categories. Effect sizes were calculated using Cramer’s V. The *p* value was set a priori at 0.05 to test two-sided significance. The Bonferroni–Holm correction was applied due to the multiple testing. Because of the small *p* values, even after adjustment based on the number of tests performed in the respective tables, the original *p* values did not change and thus the Bonferroni–Holm correction had no effect on the reported results.

To be able to depict the change in preventive health services financially, the study compared outpatient reimbursement before and during COVID-19. For this purpose, the German uniform value scale (EBM) for outpatient billing of services provided by the SHI was considered. The calculation of the compensation for the respective GOPs studied was based on the four quarters of the years 2017–2020. Changes in the reimbursement of GOPs within the quarters considered were taken into account. Time before COVID-19 represented the average costs of the years 2017, 2018, and 2019. The calculation included the multiplication of the reimbursement of the individual GOPs with the number of screenings performed, which were provided by the WIdO. If simultaneous billing of several GOPs was not possible, the mean value of the reimbursement was used for the calculation (this was the case for GOPs 01760 and 01761 as well as 01745 and 01746).

## Results

### Total utilization

In total, data from 15,833,662 AOK insured individuals in the following age categories were included: (1) 25–39 years: 1,908,846 (female), 2,013,686 (male); (2) 40–59 years: 2,731,103 (female), 2,832,784 (male); (3) 60–79 years: 2,371,561 (female), 2,113,089 (male); (4) ≥ 80 years: 1,229,909 (female), 632,684 (male).

These patients attended 11,225,261 screenings in 2017, 11,353,234 screenings in 2018, and 10,743,594 screenings in 2019. This resulted in an average of 11,107,363 attended screenings before COVID-19 (averages for the years 2017–2019). During COVID-19 (in the year 2020), the number of screenings decreased significantly by 21.46% to 8,723,989 (*p* =  < 0.001), as shown in Table 2 with the binomial test. Among individual screenings, the largest decrease in utilization was observed for the general health checkup with 45.35% less examinations (*p* =  < 0.001). With 5.99% less examinations, the smallest decrease was seen in general male cancer screening (*p* =  < 0.001). A significant decrease in utilization was also evident for the remaining GOPs. The colorectal cancer screening consultation, however, was the only exception with a significant increase of 8.92% during COVID-19 (*p* =  < 0.001). Figure 3 in Appendix provides a graphical illustration of the change in utilization of the individual screenings.

**Table 2:** Investigation of the change in utilization during compared to before COVID-19

GOP	Utilization	*z*-value	*q*	*p*
Total screenings		– 1313.88	0.298	< 0.001
Before COVID-19	11,107,363			
During COVID-19	8,723,989			
Change (%)	– 21.46%			
General female cancer screening		– 212.84	0.779	< 0.001
Before COVID-19	3,492,421			
During COVID-19	3,147,838			
Change (%)	– 9.87%			
General male cancer screening		– 64.56	0.935	< 0.001
Before COVID-19	1,027,356			
During COVID-19	965,852			
Change (%)	– 5.99%			
General health checkup		– 842.22	0.822	< 0.001
Before COVID-19	2,811,569			
During COVID-19	1,536,466			
Change (%)	– 45.35%			
Colorectal cancer screening stool test		– 257.44	0.963	< 0.001
Before COVID-19	583,324			
During COVID-19	392,478			
Change (%)	– 32.72%			
Colorectal cancer screening consultation		80.12	0.96	< 0.001
Before COVID-19	638,853			
During COVID-19	695,823			
Change (%)	8.92%			
Colonoscopy		– 43.64	0.995	< 0.001
Before COVID-19	85,341			
During COVID-19	66,919			
Change (%)	– 21.59%			
Skin cancer screening		– 375.68	0.878	< 0.001
Before COVID-19	1,931,488			
During COVID-19	1,442,453			
Change (%)	– 25.32%			
Mammography screening		– 86.23	0.966	< 0.001
Before COVID-19	537,010			
During COVID-19	476,160			
Change (%)	– 11.33%			

**Fig. 1:**
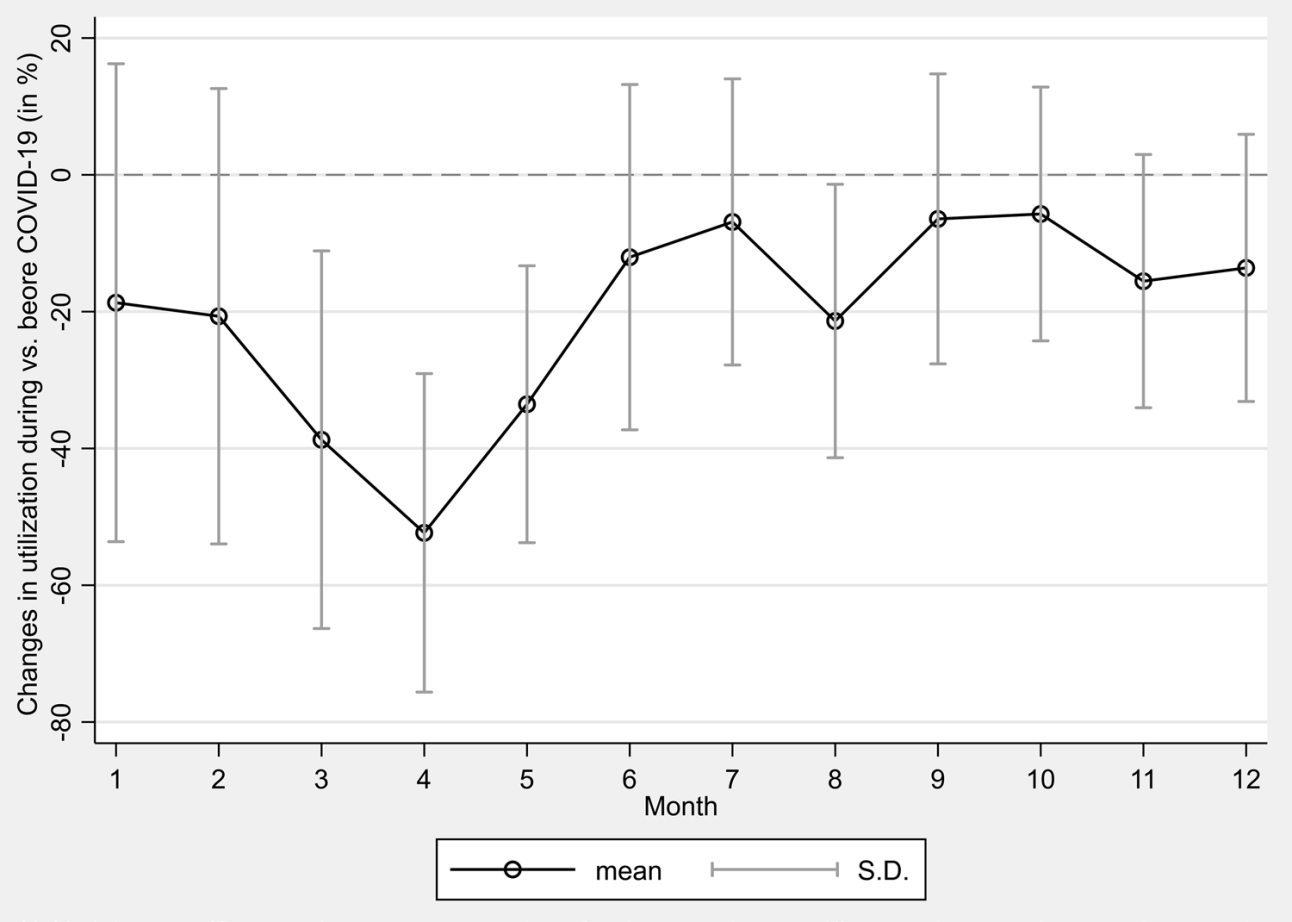
Percentage change in monthly utilization of screenings before and during COVID-19

Figure 1 shows the mean percentage change in monthly utilization of all screenings during COVID-19 compared to the utilization before COVID-19 which is indicated by the horizontal line at 0. It was found that the monthly utilization throughout 2020 was below the average utilization before COVID-19. Screening uptake was already lower in January and February 2020 (January: mean = − 18.69%, SD = 34.94%; February: mean = − 20.67%, SD = 33.29%). This decline worsened in March (mean = − 38.73%, SD = 27.60%) and reached its low point in April with a mean of 52.34% (SD = 23.28%) fewer screenings. After utilization had approached to the previous years’ levels in July (mean = − 6.88%, SD = 20.92%), another decline occurred in August (mean = − 21.37%, SD = 19.99%). Following a slight recovery in the fall, utilization dropped again in the winter, culminating in a mean percentage change of − 13.60% (SD = 19.63%) in December 2020. Both the mean percentage utilization and its SD for the months March, April, May, and August 2020 were lower than previous years’ values, indicating a sharp decline in the utilization of all screenings in these months. For the remaining months, the mean of the total screenings was also below the previous years’ values, but the large SDs that exceeded the horizontal line at 0 showed that individual screenings varied in these months, with some screenings meeting or even exceeding previous years’ levels. Overall, the mean number of screenings during 2020 did not reach the previous years’ average in any month. As the mean screening utilization did not exceed the mean screening utilization from previous years to compensate for missed screenings, no catch-up effects could be detected.

This trend is also evident in Table 3, in which monthly utilization of screenings before and during COVID-19 was further examined using the Chi-square test. The analysis showed that total screening utilization was significantly related to the respective time period (*χ*^2^(1) = 164,057, *p* =  < 0.001, *V* = 0.091). In addition to the analysis of the total screenings, claims data for individual GOPs were examined on a monthly basis. A significant association in the time course of utilization was also detected for each individual screening at the *p* < 0.001 significance level. The largest effect size was found to be *V* = 0.221 for mammography screening, followed by *V* = 0.124 for the general health checkup. Effect sizes of the other screenings were lower.

**Table 3:** Time course and screening utilization before and during COVID-19

GOP	Jan	Feb	Mar	Apr	May	Jun	Jul	Aug	Sep	Oct	Nov	Dec	*χ*^2^	*df*	*p*	*V*
Total screenings													164,057	11	< 0.001	0.091
Before COVID-19	1,215,844	1,087,095	1,125,801	942,706	1,021,052	895,074	858,610	781,113	864,040	798,702	930,417	586,910				
During COVID-19	988,555	862,381	689,788	449,273	678,610	787,345	799,532	614,217	808,407	752,995	785,821	507,065				
Change (%)	– 18.69%	– 20.67%	– 38.73%	– 52.34%	– 33.54%	– 12.04%	– 6.88%	– 21.37%	– 6.44%	– 5.72%	– 15.54%	– 13.60%				
General female cancer screening													38,226	11	< 0.001	0.076
Before COVID-19	368,213	321,953	345,948	300,294	326,851	287,816	269,587	241,576	272,378	258,782	305,337	193,687				
During COVID-19	350,615	298,934	257,862	177,870	261,730	290,600	276,434	212,374	288,067	263,247	288,355	181,750				
Change (%)	– 4.78%	– 7.15%	– 25.46%	– 40.77%	– 19.92%	+ 0.97%	+ 2.54%	– 12.09%	+ 5.76%	+ 1.73%	– 5.56%	– 6.16%				
General male cancer screening													10,208	11	< 0.001	0.072
Before COVID-19	115,109	100,819	104,417	86,940	90,377	78,537	73,672	68,792	75,677	76,624	94,064	62,328				
During COVID-19	115,458	99,903	81,800	55,984	76,537	83,474	80,437	64,102	83,665	79,455	87,148	57,889				
Change (%)	+ 0.30%	– 0.91%	– 21.66%	– 35.61%	– 15.31%	+ 6.29%	+ 9.18%	– 6.82%	+ 10.56%	+ 3.69%	– 7.35%	– 7.12%				
General health checkup													66,487	11	< 0.001	0.124
Before COVID-19	343,938	307,421	307,212	249,182	265,781	226,001	211,844	191,465	210,096	174,823	201,171	122,634				
During COVID-19	180,476	156,609	113,783	74,671	115,925	136,182	147,658	107,864	142,885	137,240	136,029	87,144				
Change (%)	– 47.53%	– 49.06%	– 62.96%	– 70.03%	– 56.38%	– 39.74%	– 30.30%	– 43.66%	– 31.99%	– 21.50%	– 32.38%	– 28.94%				
Colorectal cancer screening stool test													7613	11	< 0.001	0.088
Before COVID-19	63,503	62,237	66,886	39,554	47,195	43,163	43,644	38,762	45,425	43,557	52,241	37,158				
During COVID-19	41,272	41,261	32,711	17,387	29,073	33,332	37,684	25,835	36,548	35,114	35,955	26,306				
Change (%)	– 35.01%	– 33.70%	– 51.09%	– 56.04%	– 38.40%	– 22.78%	– 13.66%	– 33.35%	– 19.54%	– 19.38%	– 31.17%	– 29.21%				
Colorectal cancer screening consultation													6157	11	< 0.001	0.068
Before COVID-19	58,057	52,418	54,666	51,021	57,347	50,669	54,647	50,475	54,568	54,145	60,908	39,931				
During COVID-19	75,668	66,136	53,371	39,914	54,228	61,141	65,178	51,389	64,980	60,491	61,753	41,574				
Change (%)	+ 30.33%	+ 26.17%	– 2.37%	– 21.77%	– 5.44%	+ 20.67%	+ 19.27%	+ 1.81%	+ 19.08%	+ 11.72%	+ 1.39%	+ 4.11%				
Colonoscopy													731	11	< 0.001	0.069
Before COVID-19	8054	7345	7976	6971	7561	7041	7013	6711	6964	6652	7634	5419				
During COVID-19	7061	6340	5665	3694	5000	5787	6154	5040	6391	5636	5855	4296				
Change (%)	– 12.33%	– 13.68%	– 28.97%	– 47.01%	– 33.87%	– 17.81%	– 12.25%	– 24.90%	– 8.23%	– 15.27%	– 23.30%	– 20.72%				
Skin cancer screening													24,560	11	< 0.001	0.085
Before COVID-19	208,776	186,521	187,778	166,746	179,647	158,256	158,148	141,581	151,952	136,006	155,578	100,501				
During COVID-19	167,006	143,761	110,183	79,212	113,330	127,998	139,076	104,758	132,869	122,525	121,265	80,470				
Change (%)	– 20.01%	– 22.93%	– 41.32%	– 52.50%	– 36.92%	– 19.12%	– 12.06%	– 26.01%	– 12.56%	– 9.91%	– 22.06%	– 19.93%				
Mammography screening													49,629	11	< 0.001	0.221
Before COVID-19	50,194	48,381	50,918	41,998	46,292	43,590	40,055	41,751	46,981	48,113	53,484	25,253				
During COVID-19	50,999	49,437	34,413	541	22,787	48,831	46,911	42,855	53,002	49,287	49,461	27,636				
Change (%)	+ 1.60%	+ 2.18%	– 32.41%	– 98.71%	– 50.78%	+ 12.02%	+ 17.12%	+ 2.64%	+ 12.82%	+ 2.44%	– 7.52%	+ 9.44%				

The monthly change in the individual GOPs revealed a decline in utilization in most cases when comparing the before COVID-19 and during COVID-19 time horizon. The largest drop was seen in April 2020 for mammography screenings with a 98.71% decrease compared to before COVID-19. The general health checkup, with an average of 70.03% fewer utilizations in April 2020, was also affected greatly compared to the previous years’ levels. The percentage change in utilization, however, differed between screenings. While some screenings were performed less frequently, other screenings were requested more frequently in the same month than before COVID-19. For example, more colorectal cancer screening consultations were utilized each month starting in June than in the same period before COVID-19. Comparing all GOPs, demand for colorectal cancer screening consultations increased the most, whereas demand for general health checkups decreased the most.

### Differences in utilization with regard to sex and age

Beyond the consideration of the general utilization of screenings, differences due to patients’ sex and age were analyzed in more detail. Over the course of COVID-19, utilization of total screenings available to women decreased significantly by 20.56% from 7,450,140 before COVID-19 to 5,918,073 (*p* =  < 0.001) and for men by 23.28% from 3,657,223 to 2,805,916 (*p* =  < 0.001). In the age group 25–39 years, the utilization of total screenings decreased by 4.56% (*p* =  < 0.001), in the age group 40–59 years by 18.65% (*p* =  < 0.001), in the age group 60–79 years by 23.75% (*p* =  < 0.001), and for patients aged 80 years or older by 37.02% (*p* =  < 0.001). These results can be found in Table 4.

**Table 4:** Investigation of the change in utilization of all screenings before and during COVID-19 with regard to sex and age

Patient group	Utilization	*z*-value	*q*	*p*
Total screenings female		– 775.13	0.529	< 0.001
Before COVID-19	7,450,140			
During COVID-19	5,918,073			
Change (%)	– 20.56%			
Total screenings male		– 507.82	0.769	< 0.001
Before COVID-19	3,657,223			
During COVID-19	2,805,916			
Change (%)	– 23.28%			
Total screenings 25–39 years		– 56.91	0.927	< 0.001
Before COVID-19	1,149,367			
During COVID-19	1,096,952			
Change (%)	– 4.56%			
Total screenings 40–59 years		– 443.48	0.737	< 0.001
Before COVID-19	4,163,724			
During COVID-19	3,387,341			
Change (%)	– 18.65%			
Total screenings 60–79 years		– 590.80	0.719	< 0.001
Before COVID-19	4,449,227			
During COVID-19	3,392,557			
Change (%)	– 23.75%			
Total screenings > 80 years		– 449.42	0.915	< 0.001
Before COVID-19	1,345,045			
During COVID-19	847,139			
Change (%)	– 37.02%			

**Fig. 2:**
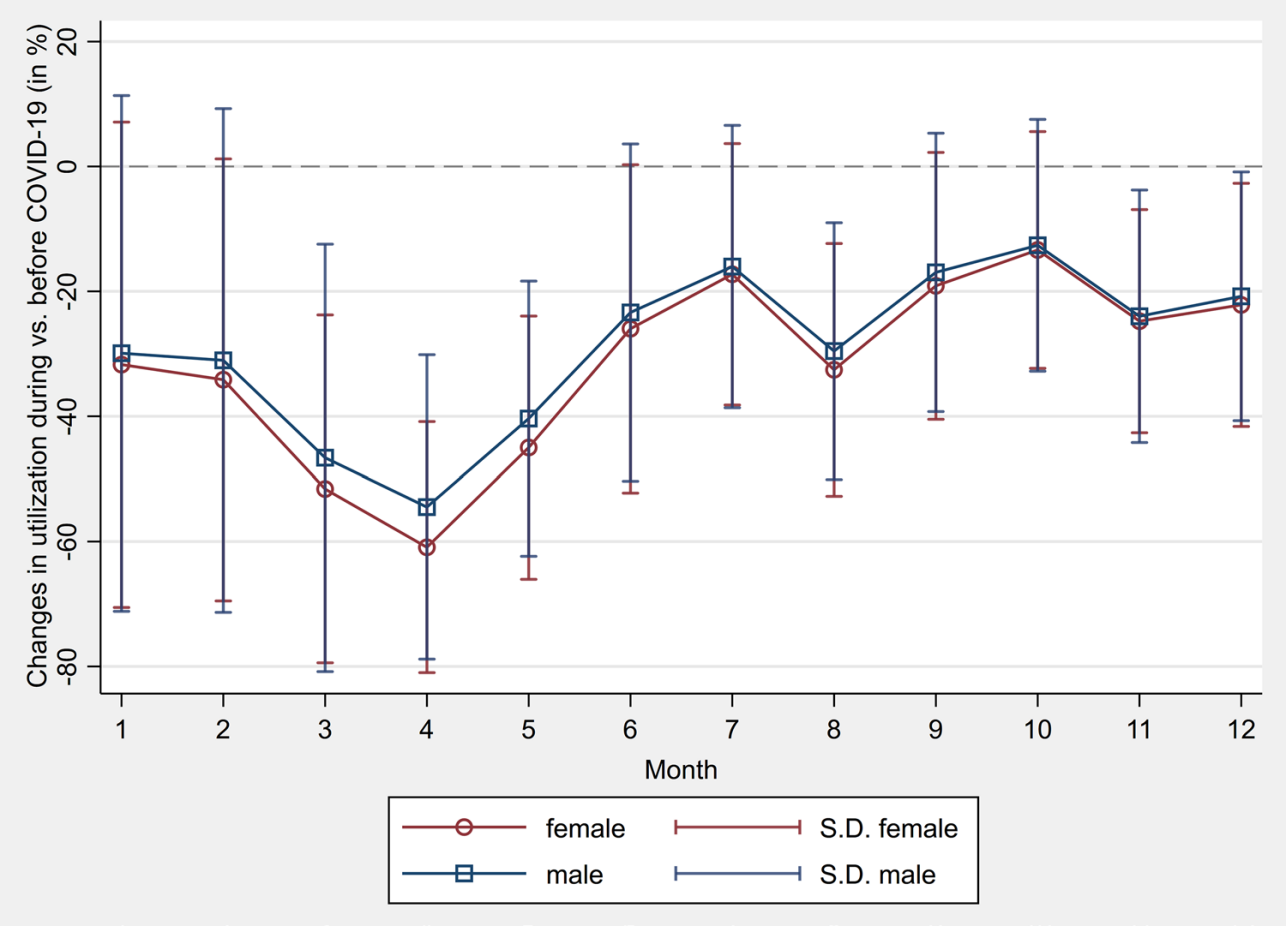
Percentage change in monthly utilization of screenings before and during COVID-19 comparing women and men

**Fig. 3:**
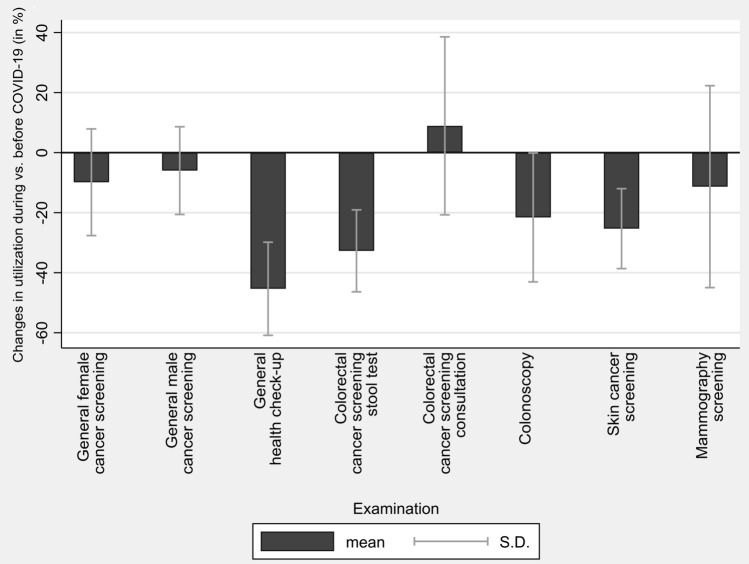
Percentage change per individual screening during COVID-19 compared to before COVID-19

The mean percentage change in the monthly utilization of screenings before and during COVID-19 was differentiated between women and men in Fig. 2. Only GOPs that could be claimed for both sexes were considered. The mean utilization of screenings has declined to a greater extent for females than for males in each month when compared to the average utilization before COVID-19. The strongest difference between the sexes was observed in April. While the mean decrease in utilization for males was 54.48% (SD = 24.34%), females utilized on average 60.92% fewer screenings (SD = 20.07%) in April compared with the previous years’ average. However, for the months July and October through December, the overlapping SD suggests that in relation to the time period before COVID-19, the difference in screening utilization between sexes became smaller. The smallest difference in the mean percentage change to the time period before COVID-19 was observed in October (female: mean = − 13.37%, SD = 18.96%; male: mean = − 12.63%, SD = 20.17%).

Furthermore, in Table 5 sex differences are examined in more detail for the entire year 2020 compared with the before COVID-19 period for screenings that were available to both women and men. The decrease in total utilization of screenings was significantly more pronounced for women than for men during COVID-19. The largest difference between sexes was found for colonoscopy with a drop of 23.32% for women and 19.64% for men (*χ*^2^(1)=21, *p* = < 0.001, *V* = 0.012). The largest decrease in examinations offered for both sexes was observed for the general health checkup with a decrease of 46.47% for women and 43.93% for men (*χ*^2^(1) = 531, *p* = < 0.001, *V* = 0.011). Colorectal cancer screening consultation increased for women and men during COVID-19 compared to before COVID-19. While women used these examinations on average 7.51% more often, the demand increased by an average of 10.72% for men compared to previous years (*χ*^2^(1) = 71, *p* = < 0.001, *V* = 0.007).

**Table 5:** Comparison of utilization between sex before and during COVID-19 for screenings available to both women and men

GOP	Female	Male	*χ*^2^	*df*	*p*	*V*
Total screenings			1088	1	< 0.001	0.010
Before COVID-19	3,420,708	2,629,867				
During COVID-19	2,294,075	1,840,064				
Change (%)	– 32.94%	– 30.03%				
General health checkup			531	1	< 0.001	0.011
Before COVID-19	1,574,858	1,236,711				
During COVID-19	842,988	693,478				
Change (%)	– 46.47%	– 43.93%				
Colorectal cancer screening stool test			168	1	< 0.001	0.013
Before COVID-19	364,352	218,973				
During COVID-19	240,043	152,435				
Change (%)	– 34.12%	– 30.39%				
Colorectal cancer screening consultation			71	1	< 0.001	0.007
Before COVID-19	358,816	280,037				
During COVID-19	385,754	310,069				
Change (%)	+ 7.51%	+ 10.72%				
Colonoscopy			21	1	< 0.001	0.012
Before COVID-19	45,048	40,293				
During COVID-19	34,541	32,378				
Change (%)	– 23.32%	– 19.64%				
Skin cancer screening			317	1	< 0.001	0.010
Before COVID-19	1,077,634	853,853				
During COVID-19	790,749	651,704				
Change (%)	– 26.62%	– 23.67%				

Detailed tables of monthly screening utilization by women and men before and during COVID-19 are provided in Appendix 2 and 3.

Differences in the change of utilization could also be observed with regard to the age of the patients, as presented in Table 6. The change in utilization of total screenings was significantly related to patient age (*χ*^2^(2) = 14,559, *p* = < 0.001, *V* = 0.038). The higher the age of the patients, the lower was the utilization of total screenings. While there were 27.11% fewer screenings in total recorded in the 40–59 years age category, the decline was 31.84% in the 60–79 years age category, and reached a maximum of 42.49% fewer screenings in the > 80 years age category. In addition, a significant decrease in utilization with increasing age was observed for all examinations, for which both women and men were eligible. Only the colorectal cancer screening stool test had a greater decrease in utilization among those aged 55–59 years (− 36.96%) than among those aged 60–79 years (− 28.18%). Nevertheless, the greatest decline was again found among those > 80 years of age (− 41.66%) (*χ*^2^(2) = 1558, *p* = < 0.001, *V* = 0.040). For colorectal cancer screening consultations, an increase in utilization during COVID-19 of 36.73% was observed among patients in the age category 55–59 years compared with before COVID-19. For patients aged 60–79 years, utilization changed by − 0.41% and patients > 80 years had a decrease in utilization of 9.02% (*χ*^2^(2) = 8354, *p* = < 0.001, *V* = 0.079). Another greater difference was found for colonoscopy, which showed a 17.87% reduction in the number of cases in the 60–79 years age category and a 54.32% reduction in the > 80 years age category (*χ*^2^(1) = 890, *p* = < 0.001, *V* = 0.076). The utilization by age groups can be found in Appendix 4 for women, and in Appendix 5 for men.

**Table 6:** Comparison of utilization between age before and during COVID-19

GOP	40–59 years	60–79 years	> 80 years	*χ*^2^	*df*	*p*	*V*
Total screenings				14,559	2	< 0.001	0.038
Before COVID-19	2,367,027	2,727,664	955,885				
During COVID-19	1,725,231	1,859,157	549,751				
Change (%)	– 27.11%	– 31.84%	– 42.49%				
General health checkup				12,244	2	< 0.001	0.053
Before COVID-19	1,205,688	1,149,165	456,716				
During COVID-19	735,828	596,004	204,634				
Change (%)	– 38.97%	– 48.14%	– 55.19%				
Colorectal cancer screening stool test^a^				1558	2	< 0.001	0.040
Before COVID-19	156,597	332,382	94,345				
During COVID-19	98,721	238,720	55,037				
Change (%)	– 36.96%	– 28.18%	– 41.66%				
Colorectal cancer screening consultation^a^				8354	2	< 0.001	0.079
Before COVID-19	181,986	363,951	92,916				
During COVID-19	248,832	362,455	84,536				
Change (%)	+ 36.73%	– 0.41%	– 9.02%				
Colonoscopy				890	1	< 0.001	0.076
Before COVID-19		76,645	8696				
During COVID-19		62,947	3972				
Change (%)		– 17.87%	– 54.32%				
Skin cancer screening				2334	2	< 0.001	0.026
Before COVID-19	822,756	805,520	303,212				
During COVID-19	641,850	599,031	201,572				
Change (%)	– 21.99%	– 25.63%	– 33.52%				

^a^Data from patients ≥ 55 years

### Health economic analysis

The results of the health economic analysis revealed notable variations in the reimbursement of preventive health services for SHI patients before and during COVID-19. The calculation in Table 7 shows that before COVID-19 a yearly mean of €274,937,166 was reimbursed for screenings and checkups. During COVID-19, reimbursement decreased to €219,378,343, resulting in a reduction of €55,558,823. The smallest decrease was noted in general male cancer screenings at €432,939, whereas the largest decrease was present in the general health checkups at €36,973,751. The only screening that was billed more often during COVID-19 was the colorectal cancer screening consultation with a change of €1,570,553.

**Table 7:** Change in outpatient reimbursement for screenings before and during COVID-19

GOP	Reimbursement before COVID-19	Reimbursement during COVID-19	Change
Total screenings	€274,937,166	€219,378,343	€– 55,558,823
General female cancer screening	€67,057,068	€59,944,056	€– 7,113,012
General male cancer screening	€15,680,030	€15,247,091	€– 432,939
General health checkup	€91,712,390	€54,738,639	€– 36,973,751
Colorectal cancer screening stool test	€4,252,667	€3,234,019	€– 1,018,648
Colorectal cancer screening consultation	€7,279,721	€8,850,274	€1,570,553
Colonoscopy	€17,697,434	€13,365,208	€– 4,332,226
Skin cancer screening	€39,550,547	€34,805,686	€– 4,744,860
Mammography screening	€31,707,309	€29,193,370	€– 2,513,940

## Discussion

### Key findings

The aim of this claims data analysis was to investigate the utilization of all preventive health services and cancer screenings offered to SHI patients in Germany before and during the COVID-19 pandemic. The study revealed two major findings. First, the utilization of total screenings decreased during the COVID-19 pandemic, but trends in utilization varied with respect to individual screenings. Second, screening utilization has developed differently among patient groups.

### Change in screening utilization

The analysis showed that the number of total screenings decreased significantly in Germany by around 21.46% in 2020 compared to before COVID-19 (*p* < 0.001). In addition, the 4-year observation period revealed a decrease of total screenings throughout the course of 2020, with the largest declines being temporally related to the lockdowns introduced in Germany. In addition to the largest change in perceived screenings in April 2020 (− 52.34%), a decrease of about 24% was also noted in ambulatory care utilization for that month, as shown by Bayindir and Schreyögg (2022) (Bayindir and Schreyögg 2022). While ambulatory care utilization returned to prior-year levels during the year (Bayindir and Schreyögg 2022), no catch-up effects were detected in our study throughout the entire year.

The decline in utilization of cancer screenings shown in our study is consistent with international findings (Doubova et al. 2021; Lantinga et al. 2021; Mantellini et al. 2020; Song et al. 2021). For example, a systematic review by Alkatout et al. (2021) and a meta-analysis by Mayo et al. (2021) reported a substantial decline in screenings worldwide (Alkatout et al. 2021; Mayo et al. 2021). Compared to the existing literature, however, our study goes beyond examining only individual screenings by providing evidence on the development of screening utilization based on the analysis of the entire prevention program in Germany and differentiates between individual patient groups.

In addition, the change in utilization before and during COVID-19 developed differently for the individual preventive health services. The highest decrease was found in general health checkup with 45.35%, followed by declines in colorectal cancer screening stool test with 32.72%, skin cancer screening with 25.32%, and colonoscopy with 21.59%. Changes were less marked in the sex-specific screenings: mammography screening, general female cancer screening, and general male cancer screening with − 11.33%, − 9.87%, and − 5.99% respectively. In contrast, colorectal cancer screening consultation, the only screening that does not involve a physical examination, increased by 8.92% in 2020. Consultation numbers as of June 2020 even surpassed the number of consultations for this screening compared to the same time period before COVID-19.

The overall negative development of patient numbers during the pandemic, both internationally and in Germany, as well as the different directions and magnitudes of changes in the utilization of the individual cancer screenings could have various causes: (1) A decrease in patient demand, which most likely is linked to the fear of an infection with SARS-CoV-2 (Hajek et al. 2021; Lazzerini et al. 2020). For example, a survey from the USA found that approximately 40.9% of respondents postponed or even avoided physician visits until June 2020 because they were concerned about COVID-19 (Czeisler et al. 2020). (2) A decrease in supply due to the suspension of services and programs, such as the interruption of breast cancer screening programs for different time periods in countries like Australia, the Netherlands, and the UK (Figueroa et al. 2021). Mammography screenings were also suspended in Germany at the beginning of the pandemic (Ärztezeitung 2020), which is reflected in our data set with a decrease of 98.71% in April 2020. At the same time, there is evidence that the likelihood of receiving appointments in outpatient practices in Germany decreased at the onset of the pandemic (Muschol and Gissel 2021), which may also have contributed to the change in case numbers for preventive health services. (3) The different multiyear eligibility of each screening may have had an influence on utilization. For example, patients are only eligible for the general health checkup every three years, which could lead to the effect that this checkup might be more likely to be postponed by patients or physicians. (4) The respective medical procedure may have had an impact on utilization. Procedures such as skin cancer screenings or colonoscopies with pronounced physical contact and a presumably more time-consuming treatment have decreased, whereas colorectal cancer screening consultations that do not require intense physical contact have increased. In addition, the ability to perform these screenings with the help of video consultations might have also led to an increase in this type of screenings. (5) High utilization of screenings prior to the pandemic could be an indicator of the perceived relevance of the respective screenings, which persisted during the pandemic. In particular, general cancer screening for women, mammography screening, and general cancer screening for men were utilized comparatively frequently by eligible patients in 2019 with 46%, 25%, and 23%, respectively (Tillmanns et al. 2021).

### Decline for women and the elderly

In addition to the general change in the utilization of individual screenings in Germany, our study also found that the utilization of screenings developed differently with regard to patient-specific characteristics such as sex and age. For screenings that can be claimed for both sexes, a significantly stronger decrease was observed for women than for men (32.94% vs. 30.03%). Structural changes in the population that occurred during the COVID-19 pandemic may have influenced utilization. For example, during the COVID-19 pandemic, the gender care gap was reflected in women having to perform more unpaid care work. This shift of time resources could have influenced women's use of medical care (Pacheco et al. 2021; Power 2020).

Patients’ age also had a significant impact on screening utilization. The older the patients, the greater the decrease in utilization of screenings. While total screenings in 2020 decreased by 27.11% for those aged 40–59 years, the decrease was greatest among patients aged 80 years and older, at 42.49%. The decline in utilization might be related to the fact that older individuals are at increased risk for a severe COVID-19 progression and have an increased risk of mortality (Romero Starke et al. 2021). In addition, the elderly are often affected by health-care inequalities as they face access barriers to health-care systems and suffer from delays in medical care (Jang and Kim 2020; Saif‐Ur‐Rahman et al. 2021). The assumption that this effect may be exacerbated during the pandemic can be supported by our findings and leads to concerns that the health status of the elderly may deteriorate (Jang and Kim 2020).

### Impact on policy and practice

Our results show that in an international comparison, a large number of state regulated preventive health services and screenings have not been performed in Germany either. The concern that cancer and other noncommunicable diseases, especially in early stages, are detected later also applies to Germany due to this trend. This circumstance could lead to a more severe disease progression and worse health outcomes for patients causing higher morbidity and mortality. This concern is supported for Germany by two studies from Jacob et al. (2021, 2022), which found that the number of cancer diagnoses decreased significantly in German practices during the COVID-19 pandemic (Jacob et al. 2021, 2022). For example, in April 2020, there were 32.0% fewer new cancer diagnoses in gynecology practices and 44.4% fewer in dermatology practices (Jacob et al. 2021).

### Economic effects

Finally, our health economic analysis revealed that the change in utilization of preventive health services during COVID-19 also led to variations in outpatient reimbursement. Our calculation showed that approximately €55,558,823 were billed less during COVID-19. A WIdO report by Tillmanns et al. (2021) presented the 2019 and 2020 spending for preventive health services. These calculations showed a difference of around €49 million. Although the WIdO calculation considered more chargeable services and only took the years 2019 and 2020 into account, it resulted in similar overall differences, supporting our findings (Tillmanns et al. 2021). In the long term, however, these saved costs will most likely be offset by the costs arising from an increased burden and duration of diseases due to delayed or omitted early detection. First evidence of cancer treatment in Germany suggests considerable decreases in cancer diagnoses and cases such as skin cancer as well as gynecologic and breast cancer during the pandemic (Jacob et al. 2021, 2022; Kaltofen et al. 2022; Kleemann et al. 2022). The total amount of the additional costs caused by this development, however, will only be quantifiable in the future.

### Practical implications

Our results show that individual screenings and patient groups underwent different shifts during the COVID-19 pandemic and that the utilization was particularly impaired among women and elderly patients. To be able to maintain public health in the long term and to be able to mitigate an increase in health-care spending, there are some practical implications in order to allocate limited medical resources in the best possible way. In the future, greater utilization of screenings should be promoted, and appropriate interventions have to be implemented by policymakers and health-care providers to support catch-up effects. Patients should be encouraged to continue using preventive health services and the safety of screenings should also be highlighted in the event of future unforeseeable developments.

Screenings that have seen the greatest decline in 2020, such as the general health checkup, colorectal cancer stool test, skin cancer screening, and colonoscopy, should be promoted the most. Furthermore, education on the utilization of cancer screening has to be tailored to individual groups of the population. In particular, access barriers for women and older patients need to be lowered and available resources should be targeted to these vulnerable groups. The use of digital applications could be promoted in the form of apps or telemedicine when suited for the respective examinations. During the COVID-19 pandemic, the use of telemedicine has increased in many areas, such as outpatient care in general medical practices or follow-up care of surgery patients, often providing satisfactory results (Knörr et al. 2022; Muschol et al. 2022). Some cancer screenings have also been supported by telehealth services (Price et al. 2022). One area that is particularly suitable for the use of telemedicine is dermatology (Trettel et al. 2018). For the screening of skin cancer, the use of artificial intelligence can also be beneficial (Sangers et al. 2021). Our study has shown that the COVID-19 related decrease in cancer screening utilization was strongly pronounced among older patients, i.e. they could be the population that benefits the most from digital health alternatives to conventional in-person screenings. When using digital health applications, it should therefore be ensured that older patients face no access barriers and that the applications are adapted to the needs and abilities of older people.

Finally, it is essential to continue monitoring the development of the utilization of screenings in the future, to timely recognize potential shifts in utilization for different patient groups, and to aim for timely reallocation of resources.

### Limitations

This study has four main limitations. First, due to data protection, access to patient data is highly regulated for research in Germany. For this reason, the study was based on an aggregated data set and an analysis of individual factors was not possible. For example, no conclusions could be drawn about the socioeconomic status of patients, although this may have had an impact on the utilization of screenings and should therefore be investigated further in future studies. In addition, besides the COVID-19 pandemic, other factors could have had an impact on the utilization of screenings in 2020, which could not be determined within the scope of the study due to the data basis. However, we consider the strong influence of the COVID-19 pandemic to be the primary driver for the development of screening utilization. Second, because of the data structure, the annual number of eligible patients for the respective screenings could not be detected. In addition, it could be the case that individual patients changed age groups during the observation period. Because of the rather large data set, however, this should not have resulted in any major bias. Third, the retrospective study design only allowed for an analysis of the past screening utilization, which is why it was not possible to make statements about future developments in screenings and prognoses about the effect of omitted screenings on future development of cancer diagnoses and disease severity within the scope of the study. Finally, the data set included a vast number of the insured population in Germany. Nevertheless, not the entire population was represented within the data set and insurance-specific patient characteristics may differ from other insured patients, especially in private health insurances.

## Conclusion

This was the first study that examined changes in the utilization of all preventive health services and cancer screenings available to SHI patients in Germany during the COVID-19 pandemic. Based on the analysis of claims data from the largest German statutory health insurance fund, it was found that the utilization of the individual screenings developed differently during the COVID-19 pandemic with an overall decline in utilization and no catch-up effects throughout 2020. This negative trend is also reflected in the international context. The patient groups of women and the elderly were particularly affected by the decline in cancer screenings. The postponement or omission of early detection of noncommunicable diseases is associated with the fear of worse health outcomes in the form of more severe disease progressions and increased mortality in the long term. At the same time, this could lead to increased health-care expenditures and a loss of productivity for the German economy. To counteract the negative trend, there is an urgent need for catch-up effects, especially for screenings, which have experienced a particularly severe reduction of utilization. To this end, resources should be targeted to encourage patients to make greater use of preventive health services and to support physicians in offering these services. To assist the delivery of screening in the future, the adoption of digital applications such as telemedicine, apps, or artificial intelligence should be expanded, as their increasing use since the onset of the pandemic has demonstrated their potential in this medical area. Only through focused collaboration between policymakers and health-care providers can the serious burdens that occurred during the COVID-19 pandemic and that extend beyond the direct impact of the pandemic be mitigated in the long term.

## Data Availability

The data sets generated and analyzed during the current study are available from the corresponding author on reasonable request.

## Appendix 1

See Fig. 3.

## Appendix 2: Comparison of monthly utilization by women before and during COVID-19

**Table:**

GOP	Jan	Feb	Mar	Apr	May	Jun	Jul	Aug	Sep	Oct	Nov	Dec	*χ*^2^	*df*	*p*	*V*
Total													122,645	11	< 0.001	0.096
Before COVID-19	790,066	712,846	750,674	635,019	695,108	610,747	581,983	527,185	587,664	543,332	628,677	386,838				
During COVID-19	655,347	573,918	463,527	292,812	461,588	546,188	548,664	419,702	558,080	517,363	540,812	340,072				
Change (%)	– 17.05%	– 19.49%	– 38.25%	– 53.89%	– 33.59%	– 10.57%	– 5.73%	– 20.39%	– 5.03%	– 4.78%	– 13.98%	– 12.09%				
General female cancer screening													38,226	11	< 0.001	0.076
Before COVID-19	368,213	321,953	345,948	300,294	326,851	287,816	269,587	241,576	272,378	258,782	305,337	193,687				
During COVID-19	350,615	298,934	257,862	177,870	261,730	290,600	276,434	212,374	288,067	263,247	288,355	181,750				
Change (%)	– 4.78%	– 7.15%	– 25.46%	– 40.77%	– 19.92%	+ 0.97%	+ 2.54%	– 12.09%	+ 5.76%	+ 1.73%	– 5.56%	– 6.16%				
General health checkup													42,127	11	< 0.001	0.132
Before COVID-19	186,685	170,274	172,293	142,020	152,815	128,925	120,522	107,949	118,418	98,171	111,638	65,150				
During COVID-19	95,634	84,365	60,939	39,575	63,992	76,705	83,967	59,792	79,887	76,999	75,057	46,076				
Change (%)	– 48.77%	– 50.45%	– 64.63%	– 72.13%	– 58.12%	– 40.50%	– 30.33%	– 44.61%	– 32.54%	– 21.57%	– 32.77%	– 29.28%				
Colorectal cancer screening stool test													5040	11	< 0.001	0.091
Before COVID-19	37,329	37,579	42,066	24,287	29,920	27,799	27,473	24,169	29,322	27,332	32,955	24,122				
During COVID-19	24,082	24,717	20,090	9941	17,572	20,672	23,014	15,416	22,952	21,960	22,794	16,833				
Change (%)	– 35.49%	– 34.23%	– 52.24%	– 59.07%	– 41.27%	– 25.64%	– 16.23%	– 36.21%	– 21.72%	– 19.65%	– 30.83%	– 30.22%				
Colorectal cancer screening consultation													4080	11	< 0.001	0.074
Before COVID-19	31,564	28,790	30,730	28,659	32,615	28,848	30,967	28,313	31,129	30,752	34,520	21,929				
During COVID-19	41,342	35,951	28,610	21,203	30,433	34,622	36,783	28,431	36,625	34,327	34,787	22,640				
Change (%)	+ 30.98%	+ 24.87%	– 6.90%	– 26.02%	– 6.69%	+ 20.02%	+ 18.78%	+ 0.42%	+ 17.66%	+ 11.62%	+ 0.77%	+ 3.24%				
Colonoscopy													493	11	< 0.001	0.079
Before COVID-19	4196	3770	4163	3670	3979	3775	3716	3561	3707	3568	4115	2827				
During COVID-19	3644	3298	2834	1791	2505	2996	3202	2592	3407	2957	3113	2202				
Change (%)	– 13.16%	– 12.52%	– 31.93%	– 51.20%	– 37.04%	– 20.64%	– 13.83%	– 27.21%	– 8.10%	– 17.12%	– 24.35%	– 22.11%				
Skin cancer screening													16,132	11	< 0.001	0.093
Before COVID-19	111,886	102,099	104,556	94,092	102,637	89,993	89,663	79,867	85,730	76,615	86,628	53,870				
During COVID-19	89,031	77,216	58,779	41,891	62,569	71,762	78,353	58,242	74,140	68,586	67,245	42,935				
Change (%)	– 20.43%	– 24.37%	– 43.78%	– 55.48%	– 39.04%	– 20.26%	– 12.61%	– 27.08%	– 13.52%	– 10.48%	– 22.37%	– 20.30%				
Mammography screening													49,629	11	< 0.001	0.221
Before COVID-19	50,194	48,381	50,918	41,998	46,292	43,590	40,055	41,751	46,981	48,113	53,484	25,253				
During COVID-19	50,999	49,437	34,413	541	22,787	48,831	46,911	42,855	53,002	49,287	49,461	27,636				
Change (%)	+ 1.60%	+ 2.18%	– 32.41%	– 98.71%	– 50.78%	+ 12.02%	+ 17.12%	+ 2.64%	+ 12.82%	+ 2.44%	– 7.52%	+ 9.44%				

## Appendix 3: Comparison of monthly utilization by men before and during COVID-19

**Table:**

GOP	Jan	Feb	Mar	Apr	May	Jun	Jul	Aug	Sep	Oct	Nov	Dec	*χ*^2^	*df*	*p*	*V*
Total													42,598	11	< 0.001	0.081
Before COVID-19	425,778	374,249	375,127	307,686	325,943	284,328	276,626	253,928	276,376	255,370	301,741	200,072				
During COVID-19	333,208	288,463	226,261	156,461	217,022	241,157	250,868	194,515	250,327	235,632	245,009	166,993				
Change (%)	– 21.74%	– 22.92%	– 39.68%	– 49.15%	– 33.42%	– 15.18%	– 9.31%	– 23.40%	– 9.43%	– 7.73%	– 18.80%	– 16.53%				
General male cancer screening													10,208	11	< 0.001	0.072
Before COVID-19	115,109	100,819	104,417	86,940	90,377	78,537	73,672	68,792	75,677	76,624	94,064	62,328				
During COVID-19	115,458	99,903	81,800	55,984	76,537	83,474	80,437	64,102	83,665	79,455	87,148	57,889				
Change (%)	+ 0.30%	– 0.91%	– 21.66%	– 35.61%	– 15.31%	+ 6.29%	+ 9.18%	– 6.82%	+ 10.55%	+ 3.70%	– 7.35%	– 7.12%				
General health checkup													24,768	11	< 0.001	0.113
Before COVID-19	157,253	137,147	134,919	107,163	112,966	97,077	91,322	83,517	91,678	76,652	89,533	57,483				
During COVID-19	84,842	72,244	52,844	35,096	51,933	59,477	63,691	48,072	62,998	60,241	60,972	41,068				
Change (%)	– 46.05%	– 47.32%	– 60.83%	– 67.25%	– 54.03%	– 38.73%	– 30.26%	– 42.44%	– 31.28%	– 21.41%	– 31.90%	– 28.56%				
Colorectal cancer screening stool test													2737	11	< 0.001	0.086
Before COVID-19	26,174	24,658	24,820	15,267	17,275	15,364	16,171	14,593	16,103	16,226	19,286	13,036				
During COVID-19	17,190	16,544	12,621	7446	11,501	12,660	14,670	10,419	13,596	13,154	13,161	9473				
Change (%)	– 34.32%	– 32.91%	– 49.15%	– 51.23%	– 33.42%	– 17.60%	– 9.28%	– 28.60%	– 15.57%	– 18.93%	– 31.76%	– 27.33%				
Colorectal cancer screening consultation													2191	11	< 0.001	0.061
Before COVID-19	26,494	23,628	23,936	22,363	24,732	21,821	23,679	22,162	23,439	23,392	26,388	18,002				
During COVID-19	34,326	30,185	24,761	18,711	23,795	26,519	28,395	22,958	28,355	26,164	26,966	18,934				
Change (%)	+ 29.56%	+ 27.75%	+ 3.45%	– 16.33%	– 3.79%	+ 21.53%	+ 19.91%	+ 3.59%	+ 20.97%	+ 11.85%	+ 2.19%	+ 5.18%				
Colonoscopy													260	11	< 0.001	0.060
Before COVID-19	3857	3575	3813	3301	3583	3266	3297	3150	3257	3084	3519	2592				
During COVID-19	3417	3042	2831	1903	2495	2791	2952	2448	2984	2679	2742	2094				
Change (%)	– 11.42%	– 14.91%	– 25.75%	– 42.34%	– 30.36%	– 14.54%	– 10.47%	– 22.29%	– 8.38%	– 13.14%	– 22.08%	– 19.20%				
Skin cancer screening													8711	11	< 0.001	0.076
Before COVID-19	96,891	84,422	83,222	72,654	77,010	68,263	68,485	61,714	66,222	59,391	68,950	46,631				
During COVID-19	77,975	66,545	51,404	37,321	50,761	56,236	60,723	46,516	58,729	53,939	54,020	37,535				
Change (%)	-19.52%	-21.18%	-38.23%	-48.63%	-34.09%	-17.62%	-11.33%	-24.63%	-11.31%	-9.18%	-21.65%	-19.51%				

## Appendix 4: Comparison of female utilization between age categories before and during COVID-19

**Table:**

GOP	25–39 years	40–59 years	60–79 years	> 80 years	*χ*^2^	*df*	*p*	*V*
Total screenings					49,935	3	< 0.001	0.061
Before COVID-19	1,149,367	2,864,384	2,658,900	777,489				
During COVID-19	1,096,952	2,356,907	2,007,544	456,670				
Change (%)	– 4.56%	– 17.72%	– 24.50%	– 41.26%				
General female cancer screening					8808	3	< 0.001	0.036
Before COVID-19	1,149,367	1,350,438	814,114	178,503				
During COVID-19	1,096,952	1,231,165	699,064	120,657				
Change (%)	– 4.56%	– 8.83%	– 14.13%	– 32.41%				
General health checkup					6270	2	< 0.001	0.051
Before COVID-19		646,276	630,480	298,102				
During COVID-19		385,588	324,562	132,838				
Change (%)		– 40.34%	– 48.52%	– 55.44%				
Colorectal cancer screening stool test^a^					1465	2	< 0.001	0.049
Before COVID-19		110,355	198,460	55,537				
During COVID-19		65,387	142,682	31,974				
Change (%)		– 40.75%	– 28.11%	– 42.43%				
Colorectal cancer screening consultation^a^					4001	2	< 0.001	0.073
Before COVID-19		111,979	192,864	53,973				
During COVID-19		146,618	190,884	48,252				
Change (%)		+ 30.93%	– 1.03%	– 10.60%				
Colonoscopy					505	1	< 0.001	0.080
Before COVID-19			40,341	4707				
During COVID-19			32,481	2060				
Change (%)			– 19.48%	– 56.24%				
Skin cancer screening					1629	2	< 0.001	0.030
Before COVID-19		450,060	440,908	186,667				
During COVID-19		347,037	322,823	120,889				
Change (%)		– 22.89%	– 26.78%	– 35.24%				
Mammography screening^a^					302	1	< 0.001	0.017
Before COVID-19		195,276	341,734					
During COVID-19		181,112	295,048					
Change (%)		– 7.25%	– 13.66%					

^a^Data from patients ≥ 55 years

## Appendix 5: Comparison of male utilization between age categories before and during COVID-19

**Table:**

GOP	40–59 years	60–79 years	> 80 years	*χ*^2^	*df*	*p*	*V*
Total screenings				3435	2	< 0.001	0.023
Before COVID-19	1,299,341	1,790,327	567,556				
During COVID-19	1,030,434	1,385,013	390,469				
Change (%)	– 20.70%	– 22.64%	– 31.20%				
General male cancer screening^a^				1710	2	< 0.001	0.029
Before COVID-19	250,983	565,715	210,657				
During COVID-19	249,833	539,288	176,731				
Change (%)	– 0.46%	– 4.67%	– 16.10%				
General health checkup				5745	2	< 0.001	0.055
Before COVID-19	559,412	518,685	158,614				
During COVID-19	350,240	271,442	71,796				
Change (%)	– 37.39%	– 47.67%	– 54.74%				
Colorectal cancer screening stool test^b^				436	2	< 0.001	0.034
Before COVID-19	46,243	133,922	38,808				
During COVID-19	33,334	96,038	23,063				
Change (%)	– 27.92%	– 28.29%	– 40.57%				
Colorectal cancer screening consultation^b^				4601	2	< 0.001	0.088
Before COVID-19	70,007	171,087	38,943				
During COVID-19	102,214	171,571	36,284				
Change (%)	+ 46.01%	+ 0.28%	– 6.83%				
Colonoscopy				384	1	< 0.001	0.073
Before COVID-19		36,305	3989				
During COVID-19		30,466	1912				
Change (%)		– 16.08%	– 52.06%				
Skin cancer screening				674	2	< 0.001	0.021
Before COVID-19	372,696	364,613	116,545				
During COVID-19	294,813	276,208	80,683				
Change (%)	– 20.90%	– 24.25%	– 30.77%				

^a^Data from patients ≥ 50 years

^b^Data from patients ≥ 55 years
